# Bœnninghausen’s Treatment of Croup

**Published:** 1889-01

**Authors:** P. P. Wells

**Affiliations:** Brooklyn


### BŒNNINGHAUSEN'S TREATMENT OF CROUP.

Practically, croup is attended with so much of suffering and danger to the patient and anxious solicitude on the part of friends and physicians, that no apology can be necessary for calling attention to a method of treatment which promises to diminish these in any degree. To the patient it is always painful, dangerous, and too often fatal, to be other than a cause of alarm to friends and dread to the intelligent and faithful practitioner. Every week it claims its victims among the young and helpless, and the skill of the best talent and learning of the profession has been wholly unavailing to avert the fatal result. Whatever, then, adds to our power over this destroyer is worthy of the profoundest attention; and any method of treatment which, after fair trial, claims to have diminished its duration or mortality, cannot be passed by, as of little moment, by intelligent and conscientious practitioners. It is notorious that the current methods, both of the old school and new, are too often followed by misfortune, to be regarded with much complacency, if indeed, it be not too great grace to the different, desultory, and often scarcely better than empirical practices in this disease, to call them *methods*.

The old school has relied, and still does, on blood-letting, emetics, mercury, fomentations, nauseating doses of different emetic substances, vesications, etc.; the new school, with better result, relies on Aconite, Hepar, Spongia, Bromine, Bi-chromate of Potash, Prot-iodide of Mercury, etc.; and yet the weekly reports of mortality are constantly adding testimony to the want of success which the ordinary use of these means is wholly unequal to prevent.

Is the ordinary success of the ordinary use of these means the best which the nature of the case admits? If so, we must be satisfied. But who dares be satisfied if a better can be obtained by other courses of practice? The profession in this country are indebted to our esteemed colleague, Dr. Carroll Dunham, of Newburgh, N. Y., for an introduction to a method, which, after protracted trial, claims a far greater success than has heretofore followed any other, both in the matters of *mortality* and *duration*. In the February number of the second vol. of the *American Homœopathic Review*, page 212, in a foot-note appended to a translation of Bœnninghausen's paper on the

"Advantages of the High Potencies," by Dr. Dunham, he gives the method of treatment of croup by this great master. In the body of the article (pp. 211, 212), the writer says: "*Of ten cases of membranous croup in children, in at least nine, the first, or the first two powders of my high potencies, when promptly administered, suffice for the most complete cure. The necessity for three powders is very rare indeed, and among three hundred cases not ten have occurred in which all the five powders, as I am accustomed to prescribe them, have had to be given.*" His five powders are, first *Acon.*, second and fourth *Hepar sulph.*, third and fifth *Spongia*, all of the 200th potency. These are given in the order of their numbers, every thirty minutes, and their administration suspended as soon as relief is manifest. So that, according to the statement above, more than two hundred and ninety out of three hundred cases of croup which were of such a character that the writer is willing to designate them as "*membranous*," were cured inside of *two hours*, and the whole three hundred were cured. There was not a failure. This is certainly a remarkable success, very remarkable, in comparison with the results of any other known treatment.*

It was the privilege of the writer of this paper to call the attention of the members of the American Institute of Homœopathy to this success, at their meeting in Philadelphia, in June, 1860, and to urge on them a trial of Dr. Bœnninghausen's method by these among other considerations.

1st. Its *simplicity*. Anybody can apply it. The most inexperienced can make no important mistake by its trial, provided he is sufficiently intelligent to suspend the administration of the powders as soon as improvement is realized. This rule cannot be neglected, if the best success is to be secured. The anxiety to do *a little more*, with the expectation of something *a little better*, should never be allowed to tempt to its violation. Confusion, vexation, and disappointment can only result from this folly.

2d. Its *safety*. To carry out the entire plan requires, even in cases where it may result in failure, only about two hours of time, the loss of which cannot seriously prejudice a case in its prospects of benefit from other treatment, if such should be necessary after the trial of this. If it does this in some de-

---

*Bœnninghausen, on seeing this statement of his method, repudiated it as to the intervals of time between the doses. In croup, as in all other cases of sickness, he was accustomed to repeat or give a new medicine, as called to do so by the symptoms of the case, and not to do either at any given stated time, determined beforehand.

gree, the fact can hardly be urged against the greater success of this method. This remark is made with a full consciousness of the importance of early appropriate treatment in every case.

3d. The method of Dr. Bœnninghausen cannot be supposed to complicate a case by medicinal symptoms of the agents employed if they are not effectual to a cure, which can hardly be said of most of the other methods employed.

4th. It is *prompt* in its efficacy, beyond any other method.

5th. If the rules given by the master are observed, the cure is more *complete*, as well as prompt.

In the twenty years' experience of the writer in the treatment of croup by the ordinary homœopathic method, it has been the common course of the disease to return on the second and third nights, with more or less severity, and till the lapse of the third night, and the then change of the cough to the catarrhal character, there was no relief to his anxiety. Exceptions to this return have been occasional, but rare. Since adopting the method of Dr. B., there has yet been *no return on the second or third nights in a single case.*

The force of these reasons was then felt by the writer to be great. Here was, undeniably, *simplicity, safety, promptness, and completeness of cure*, with *freedom from medicinal complications*, to urge the claims of the method to attention, and better than all, these claims were backed by an alleged success altogether without a parallel. But the question has risen and been asked, "Will this method cure every case of croup?" I can only reply, very likely not. I don't know, but I do know it has cured every case of croup proper in which I have employed it in a time and manner that has given me one of my sweetest delights. I have had no second or third night anxieties. But, in this connection, I may as well warn the practitioner who may be inclined to try the five powders as directed, that all *loud, coarse cough*, with or without wheezing and fever, are not *croup*, and that he may find many such which these powders will not cure, simply because they are not remedies appropriate to their treatment. These are cases much less important than true croup, and far easier of cure by *Bell.*, *Hepar sulph.*, *Phos.*, etc., as ordinarily administered. "But will these powders really cure true membranous croup after the membrane is formed?" was asked by one of my friends in Philadelphia. Then I could not answer from personal observation. Now I can. And I answer, Yes. The following cases will illustrate this:

CASE I.—Sept. 1st, 1860, quarter-past nine P. M., saw a child, four and a half years old, of fair complexion, moderately fleshy, suffering with symptoms of croup. Breathing difficult, with loud, sawing wheeze, cough frequent and violent, with the characteristic sound of croup; great restlessness. Expression of face anxious. The whole surface covered with profuse hot perspiration; pulse, 130 per minute, full and hard. *Breath offensive.* I prepared the five Bœnninghausen powders, gave No. 1, and in half an hour No. 2, and then No. 3, when the child became more tranquil, the skin cooler, less perspiration, cough less frequent, the peculiar sound of the respiration gone, and the child was drowsy and wanted to go to bed. He had been in his mother's lap. I supposed the battle was won, and left with directions if the breathing became embarrassed again to give the other powders at half-hour intervals.

At two A. M. I was called again, the child being reported much worse, and the parents thought him dying. I found him breathing with great difficulty—respiration loud, with the peculiar wheeze of croup—cough frequent, violent, and dry, with strong croup tone, perspiration profuse and hot, great anxiety and restlessness. Inspection of the throat showed a patch of membranous deposit on the left tonsil as large as my finger nail. The mother had given powders 4 and 5 with no relief. I gave Spongia$^{200}$ (Lehrman's), with partial relief *for a time*, when the symptoms returned, and Spongia gave no relief. I then gave Bromine, from four A. M. to seven A. M., at half-hour intervals, the only result of which was an increase in the frequency of the pulse and heat of skin. No relief of respiration or cough. I determined to repeat *the "five powders,"* looking on the case as a second attack or relapse, the result of cold feet and legs, when he awoke at two o'clock, from exposure during sleep—a fact which I now learned for the first time. The three first were given and all symptoms of croup disappeared in less than two hours, and did not return. There was a slight catarrhal cough which yielded to Phos.$^{200}$. At ten A. M. the membranous patch was evidently thinner than when examined in the night, and at five P. M. had disappeared entirely, leaving the surface of the tonsils and fauces generally deep-red, and a good deal swollen. This inflammation soon disappeared under the influence of the remedies above named.

Were not here *two attacks* of croup? My first impression was that the powders had failed. When I learned of the cold feet and legs, I took another view of the case and treated it as a new attack, with the above result. But, suppose there was only one, and the symptoms at two o'clock A. M. were only a recurrence of those *seemingly* subdued at eleven P. M., what is the objection to a repetition of the series of powders if a case should occur where the first five should prove inadequate to the cure? I have not the slightest doubt I should have lost my case if I had adhered to my first impression of the failure of the treatment. The case grew worse rather than better under Bromine (and the cases this remedy has not cured, after the failure of the ordinary remedies, nothing has cured in my practice). But, on giving the five magical powders the second time, or rather three of them, the result was even more prompt and satisfactory then when given the first time. Would this have been the case if the second attack had been only a recurrence of partially subdued symptoms? Do not such recurrences come with increased inveteracy? as is so often seen in cases of hydrocephalus.

In this case the membrane was perfectly apparent on the tonsils. Indeed the whole train of symptoms, without this, was indicative of this form of the disease, but the sight of the deposit places the matter beyond doubt.

It is worthy of remark that powders 4 and 5, Hepar and Spongia, given by the mother, after the relapse, previous to my seeing the little patient the second time, were followed by no mitigation of the symptoms; but that *Aconite*, *Hepar*, and Spongia were afterward promptly efficacious, even more so than at the first. From this it would appear that the *order of sequence* of the administration of the powders is not a matter of indifference. It may prove to be essential to success.

CASE II.—The following case has been kindly furnished me by my esteemed friend, Dr. Carroll Dunham, of Newburgh, N. Y. It further and beautifully illustrates the affirmative answer given above in relation to the cure of membranous croup, and also answers the question as to a repetition of the series of powders in any case.

On the evening of January 24th, I received a message to the effect that a little boy, aged eighteen months, fat and healthy, was slightly feverish, and somewhat hoarse. I was requested to send some medicine. I sent a powder of Acon.$^{12}$, mentioning to the messenger that croup might perhaps be threatening and requesting to be sent for on the first indications of that disease. The next morning I was told that the child was not much better, and was requested to visit it in the course of the day. I went immediately. As soon as the hall-door was opened, I heard the hoarse, ringing respiration of the child, which was in the second-story, and which I found sitting up in its crib, with an expression of great anguish, breathing at the rate of 35 in the minute and with great labor. There was but little cough; occasionally an effort which resulted in a hoarse dry bark, but which was immediately suppressed, apparently because it interfered with respiration. The face was turgid and of a purple hue. The hands were frequently applied convulsively to the larynx, but as a general thing the child was quiet, looking with pitiful, appealing eyes to the bystander, as if for aid. The skin was hot and dry except on the forehead, which was moist and cool, pulse hard, not full, 130. On saying to the mother, "the child is exceeding ill," I was told, "he has been as bad if not worse all night." He had vomited once, about an hour before my arrival, bringing up a small piece of tough membrane.

Here was a case of membranous croup, of great severity, which had been in full blast at least twelve hours before I was called to it; in which the purple, turgid face and the exhausted aspect of the child, showed that the powers of life had already begun to fail under the imperfect decarbonization of the blood. Considering the gravity of the case, and its long duration before treatment was begun, I hesitated to give the powders recommended by Bœnninghausen, but gave at once Bromine$^{1}$, centesimal, in water, a teaspoonful of the solution every fifteen minutes.

At the end of two hours, the child was in no respect better—the pulse was weaker and more frequent; there had been no relief for an instant to the labored character of the respiration, which numbered now 40 in the minute. I gave Hepar sulph.$^{2}$ trit., alternately with the Bromine. At the end of two hours there was still no change for the better; the disease was steadily advancing, as it seemed, to a fatal termination. Already it had reached a point at which I have seen both Guersant and Trousseau at the Enfans Malades refuse to perform tracheotomy, on the ground that the disease had, by its long duration, so prevented oxygenation of the blood and consequent renovation of tissues that a favorable issue could not be hoped for. I determined now to give the Bœnninghausen powders; waiting, therefore, a half-hour from the time at which the last dose of the Hepar was given, I gave a powder of Acon.$^{200}$, to be followed at intervals of a half-hour by Hepar$^{200}$, Spongia$^{200}$, and this series repeated (the method indicated in a foot-note to my translation of Bœnninghausen's article, *American Homœopathic Review*, vol. 2, p. 212). It was now five P. M., a time of day after which croup generally begins to be aggravated. At seven o'clock the child was greatly relieved, respiration 30 in the minute, much less labored, the sound softer, cough rather more frequent and somewhat loose in sound. I left a second series of the powders to be taken at intervals of one hour. The child slept at eleven P. M., and at intervals during the night, and the next morning was so much better that it seemed unnecessary to give more medicine, although I left a series of the powders to be given in case of a relapse. They were not given, however. The child recovered rapidly without relapse or sequelæ of any kind, and on the fifth day was as well as usual.

This was unquestionably the most severe case of croup that I have ever seen recover in this or any country. Judging from my experience with Bromine and Hepar in other cases, I have no hesitation in saying that, not acting more evidently and more promptly than they appeared to do in this case, nothing whatever was to be hoped for from them. In croup, if they act benefically at all, they do so promptly. It seems impossible, therefore, to ascribe the recovery of this child in any degree to these remedies, or to deny the curative action of the Bœnninghausen powders.

CASE III.—May 7th, 1860. At ten o'clock A. M. saw M. F., two and one-half years old, who had been suffering from severe catarrhal symptoms with sore throat the last four days. At four o'clock this morning she awoke her parents by her choking, strangling, and loud croup cough, for which she had had from them, Aconite and Spongia in alternation every half-hour without relief. The skin was hot and rather moist, the face red, the eyes staring, expression anxious, breathing loud, wheezing, and labored, cough frequent and loud, with the characteristic croup tone ; swallowing difficult and evidently painful, with great restlessness. I gave the five powders as directed by Bœnninghausen, with instruction to suspend their administration on the appearance of relief of the symptoms. She took the first three, and the relief was so apparent that, in accordance with instructions, she was left to their action. I saw her again at five P. M. She was playing on the floor, and apparently well of all her troubles except the difficulty of swallowing, which was removed promptly by Bell.$^{200}$. The next morning she was dismissed cured.

It is hardly necessary to remark that the worst form of croup is very often preceded by a catarrhal stage like that by which this case was ushered in, is of the most obstinate nature, and not unfreqently fatal.

CASE IV.—The following case, furnished by my friend and neighbor, Dr. R. C. Moffat, is given to illustrate the fact that this method is curative, and triumphantly so, even in cases where massive doses of the same medicines have failed.

Called in the morning to attend a child attacked during the night with croupy cough and restlessness, which were represented as severe. The child was nine months old, large, healthful, and vigorous. The usual remission had occurred, and then presented nothing but the usual loose cough. I prescribed *Aconite* and *Spongia*, one drop each, in two-thirds of a tumbler of water, a teaspoonful alternately every hour and a half, the intervals to be shortened to half an hour if necessary during the night.

The next day the report was, a very bad night; the cough, fever, and restlessness worse than the night before, and still the remission of the morning was very marked, only a loose cough with hardly any croupy tone to it. Thinking the family (a nervous one) had exaggerated their report, I gave directions —*Hepar*³, every hour and a half till the cough should lighten in the evening, and then, if the fever, etc., returned, as on the previous night, *Acon. and Spongia, stronger than before, every half-hour.*

Making an early morning call, I found the night had been worse than either of the preceding, and the cough was distinctly croupy, though the respiration, etc., were easy.

I gave *Sac. Lac.* for five hours (from ten A. M. till three P. M.), and then set out Bœnninghausen's prescription—Acon., Hep., Spong., Hep., Spong., each 200th, at half-hourly intervals, with directions to call me in the night, if disturbed.

At my morning call I found the child had slept well all night, and it required no treatment subsequently.

CASE V.—The following case in the practice of my friend and neighbor, Dr. J. Barker, illustrates the efficacy of the method in a class of cases almost always fatal when treated by any of the ordinary courses of practice.

In the fall of 1859, W. L., a child six months of age, while suffering from a severe attack of scarlet fever, was seized with marked symptoms of croup. Regarding the complication as serious, and not being satisfied with the usual remedies in similar cases, I resorted, for the first time, to Bœnninghausen's treatment for croup, viz.: Aconite, Hepar, and Spongia, each of the 200th potency. The powders were numbered in the following order: Aconite 1, Hepar 2 and 4, Spongia 3 and 5, and directed to be given with an interval of thirty minutes in the order marked. After the expiration of three hours I again saw my patient, and found him almost wholly relieved from all croupy symptoms. The attendants expressed great surprise at the prompt action of the remedies, assuring me that after giving three powders the disease at once gave way. I have since used the same prescription with most marked success in other cases of croup, and am convinced that no other treatment in my hands has proved equally efficient.

This case occurred during a severe anginose affection of the throat, and interested me much when first related to me by my friend, as it was then the first case of recovery from a similar condition I had known. In my previous experience all such cases had terminated fatally.

Case VI.—The following case in the practice of my friend, Dr. Morrill, of this city, exhibits the efficacy of the Bœnninghausen method after a continuance of the croup two days without relief from massive doses of Ipecac and Squills, a common allopathic treatment of the disease, and also after a failure of lower attenuations of Aconite and Spongia.

Mary Kelly, aged two and a half years, took a severe cold a day or two before leaving Montreal on her way to this city. The first night from home, had a slight attack of croup, which her mother treated with Syrup of Ipecac and Squills. She appeared better in the morning, but the cough continuing, the same medicine was repeated.

The next night she had a second attack, more severe than the first. The mother administered Ipecac and Squills, and these not helping the child I was sent for. The symptoms of croup were very decided. Gave Aconite$^{1}$, in alternation with Spongia$^{3}$, which soon gave relief, and the child slept quietly till morning.

The next night I was again sent for about eleven o'clock, and found the child in apparently the advanced stages of croup. There was the ringing, metallic cough—the stridulous breathing, and great difficulty of inspiration at all. The pulse was very quick, and the skin hot and very moist. Appearances were very unpromising; gave the five powders of 200th, Acon., then Hepar, then Spong., in alternation. In about an hour the child became easier, and soon after it took the fifth powder, dropped asleep. I called the next day and found the patient quite well. It has had no return of the disease since.

Comments:—These six cases will illustrate the action of these remedies in the treatment of croup. That they were cases of true croup was not a question of doubt. They were treated before the reception of Bœnninghausen's repudiation of the half-hour interval between the doses. And, nothwithstanding this error, which no doubt was a violation of a fundamental rule which dominates all sound homœopathic practice, they were cured, promptly, permanently, and perfectly, and in each case there was nothing of the return of symptoms to which we had all been accustomed when we had treated croup with our accustomed numbers in our heretofore accustomed method of administration. This was a great surprise to each of us, and the question could hardly fail to arise, How could so perfect and prompt cures follow so marked a violation of a rule of practice so important as this: "*No repetion of doses or change of medicine while improvement progresses from that already given.*"

The question will ask itself now, when we read the report given above, and we can see but one answer to it.

These cases were treated previous to 1859, when we all had had but small experience with dynamized numbers above the thirtieth. It will be remembered at this time, we had been taught, at least many of us, that acute cases required for their speediest and best cure *low* numbers, while chronic were best treated by *high*. This false rule was very generally accepted and acted on by those who at that time were leaders in the homœopathic school of practice. And sicknesses of rapid progress in their distinctive processes, like croup, were treated oftener than otherwise by numbers not above the third, and often by those lower, even by the crude drug; and with croup, where this was overcome by these low dynamizations, the painful and anxious experiences of the second and third nights were always present. The thought then was that it was the *matter* of the drug which cured, and the third, if it failed to give relief, it was because more matter of the drug than was contained in this number was required for relief, and more was given, even to very considerable quantities of the crude drug, and then, if the case terminated fatally, why, was not all done that could be? Were not the accepted remedies for croup all given, and in sufficient quantities to cure if this had been possible?

Yes, "all" was done but the right thing, and the wrong was overdone, and hence the fatal termination of the case. This was the common practice and experience previous to the publication of what was supposed to be Bœnninghausen's method with croup. This publication contained the error as to time between the doses, which we have pointed out, and yet cases were cured as they had not been by the previous practice with low numbers and crude drugs. And why? This question could not fail to ask itself, and the only answer to it we can see is because the curatives were given in *higher dynamizations*.

The results of the treatment of these six cases demonstrate, we think, conclusively, several truths of great practical importance. 1. That it is not the *matter* of the drug which *cures*, because here, when the doses given which cured so much more speedily and permanently must, if they contained any *drug matter* at all, have been in much *less quantity* than in those given in the previous practice with low numbers and the crude drug. 2. These cases, and a multitude of others similar to them, demonstrate the greater curing power in the dynamized than in the crude drug. And, 3. The superior curing effects of these high potencies over the third and lower demonstrate an increase of curing power by continuing the process of dynamization. The third number is, no doubt, a dynamic specimen of a drug power; but when the dynamizing process has been carried, as in the case of the medicines used in these cures, to the 200th, the result has been, and is, increased curing power in the higher number when its action is compared with that of the third or lower dynamization. 4. The falsehood of the rule formerly taught—low numbers in treating acute diseases and high in chronic—is fully demonstrated. The superior results of the treatment of croup by Bœnninghausen's powders as compared with the experience of treatment with lower numbers can only be accounted for by the fact of the greater curing power in the higher dynamizations. And it is not a little interesting that in these six cases and in a multitude of similar ones these superior cures were wrought, notwithstanding the 200th dynamization employed in them was handicapped by so important a violation of law in the matter of time between the doses. The increase of curing power was equal to the cure, and would have it in spite of so important a violation of law.*

P. P. WELLS.

BROOKLYN, November 29th, 1888.

---

A COUGH SYMPTOM:—Cough, with hæmoptysis and feeble, weak heart action; deep, violent in evening and night, without waking; renewed by change to cold weather and by cold winds. Expectoration pale, sweetish, unpleasant tasting, at times difficult, renewed same as cough—Lycopus-virg.

* This paper upon croup was published some twenty-eight years ago by Dr. Wells; he has recently revised and added to it. We republish it by request of some of our readers. Although this method of Bœnninghausen's has such high indorsement, we must remind our readers that the practice is open to grave errors, and violates the cardinal principles of homœopathic philosophy. Homœopathic practice treats *symptoms*, not diseases, nor does it alternate remedies. These three remedies are invaluable in treating cases of croup, but they should be prescribed, as in other diseases, only when indicated by the patient's symptoms.—EDITORS.

